# Exploring perspectives of supporting the process of dying, death and bereavement among critical care staff: A multidisciplinary, qualitative approach

**DOI:** 10.1177/17511437241308672

**Published:** 2025-01-03

**Authors:** Elsa Joyce, Suzanne Guerin, Lindi Synman, Melanie Ryberg

**Affiliations:** 1 UCD School of Psychology, University College Dublin, Dublin, Ireland; 2 Tallaght University Hospital, Dublin, Ireland; 3 School of Medicine, Trinity College Dublin, Dublin, Ireland

**Keywords:** Bereavement, dying, end of life, palliative care, critical care, ICU, families, staff perspectives, qualitative

## Abstract

**Background:**

Dying and death in critical care settings can have particularly negative implications for the bereavement experience of family members, family interaction and the wellbeing of critical care staff. This study explored critical care staff perspectives of dying, death and bereavement in this context, and their role related to patients and their families, adopting a multidisciplinary perspective.

**Method:**

This study employed a descriptive exploratory qualitative design, using reflexive thematic analysis to interpret the data. Semi-structured interviews were conducted with 15 critical care staff from hospitals in the Republic of Ireland. Most participants were female (*n* = 11), with four male participants. Professional disciplines included nursing, dietetics, physiotherapy, anaesthesiology and medicine.

**Results:**

Key findings included supporting a ‘nice death’ for patients and their families, the challenges critical care staff experience, the need for better supports in critical care, and the need for change in current bereavement support provision given the diversity evident in the modern Irish population.

**Conclusion:**

This study suggests that the unique challenges faced by staff and families throughout the dying process may benefit from the development of additional psychological, educational, and infrastructural supports. Inconsistencies in supports across critical care units in Ireland were also identified. Future research should complement the current study and examine family members’ experience of the dying process in critical care and their perspectives on supports provided.

## Introduction

International research reports adult intensive care unit (ICU) mortality rates of between 7.9% and 29% across the UK, US, Australia, and New Zealand,^1^ with an approximate 14% mortality rate observed in Ireland.^2^ Research with families highlighted aspects of these settings, including the impact of multibed wards (compared to private rooms), the noise and intimidation of machines and treatments, and the lack of comfortable facilities for visitors, while participants have described these settings as noisy, ‘*hectic*’ and ‘*busy*’^3^ (p. 35), with staff expressing similar sentiments.^4^ The disruptive and acutely stressful nature of this environment can negatively impact family access to their loved one, their connections with them and the stress of the experience, as well as the wellbeing of critical care staff.^1,5^

Bereavement is the period of grief experienced by an individual following the death of someone close to them.^6^ As such, bereavement is an intrinsic part of critical care settings and practice.^1^ Research highlights the difficulties experienced when a family member dies in ICU,^7^ which includes high levels of grief that impacts physical, mental, emotional and social health.^8^ Studies have also shown the negative emotional impact that dying, death, and bereavement in the ICU has on critical care nurses including secondary traumatic stress and avoidance^9^ and emotional distress.^5,10^ Previous research has demonstrated the distress ICU nurses experience in relation to futile care and what are considered to be ‘bad deaths’.^11-13^

Recent evidence established the importance of staff providing bereavement support to families *throughout* the dying process.^1^ However, the international research suggests a lack of sufficient and consistent bereavement support for families across the US,^14,15^ the UK,^7^ Denmark,^16^ Australia and New Zealand,^17,18^ concluding that bereavement support in critical care settings is inadequate. There is little bereavement support available to families in ICU settings in the time following death, with much of the follow-up supports relying on external services.^7,8,14^ Research proposes that developing consistent, structured bereavement support guidelines and practices would help to support critical care staff in supporting families.^19,20^ However, despite this, international ICU bereavement care procedures for critical care staff are lacking.^19^

Qualitative studies of bereavement support in ICU settings focuses on nurses’ perspectives.^4,17,21^ In an Irish context, one qualitative study explored bereavement support and the role of staff in caring for bereaved families.^22^ However, this research explored the perspectives of all staff involved in the dying process, rather than the views of ICU staff specifically. Recommendations for improving overall bereavement care in Ireland have been suggested, such as delivering training in bereavement support, particularly complicated grief and developing a national adult bereavement network.^23^ Despite this, little research has been conducted in Ireland exploring bereavement support specific to ICU, nor the perspectives of the multidisciplinary teams (MDTs) involved in caring for patients and their families.

The present study aims to explore the perspectives of critical care staff of supporting the process of dying, death and bereavement in critical care settings in Ireland, and their role in this context relating to patients and their families.

## Method

This study employed a descriptive exploratory design, conducting qualitative semi-structured interviews with critical care staff on dying, death, and bereavement across Irish hospitals. The research team included two researchers, a clinical psychologist embedded in a critical care team, and an anaesthesiologist with a special interest in intensive care medicine. The lead researcher had no previous interaction with critical care staff; thus had no preconceptions about aspects of care discussed. The differing perspectives of the research team allowed for a rich exploration of the data and reflection on the findings.

### Participants

An initial target of 15–20 participants was determined based on previous studies examining experiences of critical care staff^4,24,25^ and sample size recommendations for reflexive thematic analysis.^26^ Participants were recruited using purposive sampling through professional bodies and special interest groups, complemented by snowball sampling. Social media platforms were used to encourage participation. Recruitment and data collection took place from 24 March 2022 to 14 May 2022.

A sample of critical care staff (*n* = 15) from five different Irish hospitals, ranging in experience from 6 months to 23 years, were interviewed about their experience of dying, death and bereavement in critical care, and their role in bereavement support. The participant group were majority female and were from several different professional disciplines (Table 1).

**Table 1.** Frequencies of demographic information.

Participants	ID	*n* (%)
Gender		
Female		11 (73.3%)
Male		4 (36.7%)
Professional discipline		
Nursing^a^	NP	7 (46.7%)
Anaesthesiology^b^	MP	3 (20.0%)
Dietetics	AHP	2 (13.3%)
Medicine^c^	MP	2 (13.3%)
Physiotherapy	AHP	1 (6.7%)

NP: nursing professional; MP: medical professional; AHP: allied health professional.

a Including staff nurses, nurse specialists, nurse managers.

b Including anaesthesiologists, registrars in anaesthesiology.

c Including consultants, medical doctors.

### Procedure

The semi-structured interviews followed a schedule designed by the research team (provided as part of the Supplemental Material), which aimed to capture elements of the experience of ICU staff and their role in the dying process, bereavement support practices and recommendations for improving bereavement support in critical care. This schedule was first piloted with a volunteer medical professional to ensure efficacy of the online interview process. It was then piloted on a second specialist volunteer working in critical care, not included in the final participant pool, to confirm suitability of interview questions.

Participants were invited by professional bodies, special interest groups and through social media, to contact the research team if they wished to participate in the study. Upon doing so, an information sheet was sent to participants via email. Following this, their informed consent was obtained prior to interview.

All individual interviews were conducted in a conversational style by the same researcher. Data were collected by digital audio recording over the online platform, Zoom. These recordings were then transcribed verbatim and any identifiable information was replaced with anonymised identifiers for example, [hospital name]. Participants were informed that they could withdraw their consent until their data had been de-identified, approximately 2 weeks after interview.

Reflexive thematic analysis with an inductive approach^27^ was used to analyse the data. As per Braun and Clarke’s^28^ process of thematic analysis, transcribed interviews were systematically coded by the lead researcher, where each line was coded relative to its significance and content. Another researcher shadowed a third of participant interviews and coding. Descriptive themes were developed and from these, analytical themes were produced where they may not have been explicitly found in the original material. Two authors conducted a ‘sense’ check on candidate themes produced by comparing them to a third of the interview transcripts. Themes were then discussed by the whole research team to allow the identified key findings to be synthesised and finalised.

An example of reflexive thematic development in this study can be demonstrated by the theme that refers to a ‘nice death’. While the research team recognised the existing concept of a ‘good death’ in critical care research, the lead researcher felt the concept of a ‘nice death’ represented perspectives evidenced in the study more broadly. However, this distinction was not made until the final process of analysis where themes were synthesised.

To note finally, in results where quotes are presented, individual identifiers are not provided to avoid identification. However, the broad profession of the participant is indicated, as per IDs in Table 1.

This study was approved by the Taught Masters Research Ethics Committee - Psychology at University College Dublin, as any possible concerns were acknowledged and clearly handled within the study (approval number 2021-23).

## Results

Themes developed from the data highlighted four key findings that clustered around topics reflecting the focus of the study (See Table 2). These findings are reported in detail below, and Table 3 in the Supplemental Material provides additional illustrative quotes alongside the themes and subthemes.

**Table 2.** Overview of key findings, themes and subthemes.

Key findings	Themes	Subthemes
The critical care environment undermines staff in supporting a ‘nice death’	Staff want to provide a ‘nice death’ to patients and their families	Want to ensure the patient and family’s wishes are adhered to
		The importance of communication in providing a ‘nice death’
		Managing expectations is crucial to providing a ‘nice death’
	Staff have a role ensuring the comfort of the patient	Inclusion of palliative care and its importance
	‘Huge’ supportive role for family	Role in supporting religious practices
		Role in providing psychological support
	Role in deciding and initiating dying process	Importance of not putting responsibility of decision on families
	Environmental and organisational barriers get in the way of staff providing the care they feel families deserve at end of life	Organisational challenges to providing care to families
		Environmental challenges to providing care to families
The desire to support a ‘nice death’ poses distinct challenges for staff	Staff struggle with moral dilemmas related to dying and death in the ICU	
	Staff experience of supporting the dying process differs depending on death trajectory	
	Burnout and psychological impact of critical care ‘very real’ for staff	
Enduring the ‘tough’ nature of dying in critical care requires better supports for staff and families	The ‘tough’ nature of death in ICU and its impact on staff demands additional support for ICU staff	
	Staff are sometimes not aware of the resources available to themselves or families internal/external to hospital	
	Supports and resources for staff and family is inconsistent, insufficient, and not specific enough	
	Formalised bereavement support training and education could assist staff to provide a ‘nice death’ to families	
	Debriefing is an important support for staff	
	Families need additional support due to the nature of death in ICU	Families need space to grieve
		Psychological support is hugely important for families and staff
		There is a need for follow-up for families after death in ICU
	COVID highlighted the lack of resources for staff	
	COVID disrupted the bereavement process for families and staff	
Staff recognise the need for bereavement supports to reflect a multicultural Ireland	The evolving multicultural diversity of Ireland changed staff members’ ability to understand family’s needs	There is a need for cultural/religious education of staff
	Bereavement supports need to better cater to the evolving multicultural diversity of Ireland	Chaplaincy is the most common support for families

### The critical care environment undermines staff in supporting a ‘nice death’

The first key finding reflected themes around providing a ‘nice death’, and the challenges in the critical care environment that disrupt this. The choice of the phrase ‘nice death’ represents a distinct meaning, identified through participants’ discussions of supporting both a ‘*good death*’ and a ‘*nice death*’ and the researcher’s interpretation of the data. The use of the word ‘*nice*’ by staff was interpreted to reflect a broader concept in critical care that encompassed creating a more positive experience of the dying process for families in ICU and seemed separate to the concept of a *‘*good death*’*. Staff described the desire to respect patients’ families, with one participant describing it as ‘*recognition of the love they have for their loved one*’.

A ‘nice death’ as suggested by the findings involves catering to the needs of the patient and family in a personalised way. Staff described going beyond the scope of care and into the patient’s personhood by conducting individualised care practices. Staff reported a desire to ‘*have everything as nice as [they] possibly could*’ for patients and their families at end-of-life. Staff felt they serve a ‘*huge*’ supportive role for families, including taking care of their emotional, psychological and spiritual needs, with an additional theme reflecting their role in decision-making and initiating the dying process, and a related subtheme highlighting the importance of not putting the responsibility for the decision on families. Practices to support a ‘nice death’ included brushing the patient’s hair, turning off monitors on machinery and ensuring the room is tidy. Ensuring patient comfort was described as important and is reflected in the expressed desire to involve the palliative care team in the dying process. As one participant reported, ‘*We probably could be better at involving palliative care services as well. It’s something we don’t tend to do all that often in ICU*’ (MP).

Subthemes of supporting a ‘nice death’ included managing the family’s expectations throughout the dying process and ensuring the patient’s wishes are adhered to. In addition, a subtheme emphasised the importance of ‘*soft*’ communication and its essential role in providing a ‘nice death’ for patients and their families, with a specialist nurse saying, ‘*Bad communication leads to a bad death, not just for the patient, but [importantly] for the family*’ (NP). Other practices described to support a ‘nice death’ and cater to the spiritual needs of the patient and family included facilitating a blessing by a priest or displaying Catholic artefacts around the bedside of the patient.

Staff described however, the presence of environmental and organisational barriers that hinder them in providing the care they believe patients and their families deserve at end-of-life. ICU was described as ‘*not the ideal environment to die in*’. Staff felt that the critical care environment was a distraction to families and added to their traumatic experience. Infrastructural and organisational challenges to supporting families included restrictions in the availability of bereavement support staff after hours, lack of practical resources for families and a lack of family rooms, which were seen as offering a private space where end of life discussions could take place. Staff felt their desire to support a *‘*nice death’ was disrupted by these factors, with one participant noting, ‘*It’s really hard* . . . *to provide holistic, person-centred, kind of nice end of life care in like a setting replete with depersonalising devices*’ (NP).

### The desire to support a ‘nice death’ poses distinct challenges for staff

Staff members’ experience of supporting the dying process differs depending on the death trajectory of a patient. Staff discussed challenges that arise from their desire to provide a ‘nice death’, including professional and moral conflicts arising from care decisions that disrupt them from fulfilling their patient’s wishes, and the diverging goals of disciplines within the MDT:

We’re fighting all the time [with medics] to try and get somebody comfortable when you know, deep down, that they’re not going to survive this and they’re not going to. . and you want them to be comfortable like. (NP)

Staff discussed the need to appropriately withdraw treatment at the ‘*point of futility*’ to ensure patient comfort and to allow for a ‘nice death’. Another ethical challenge related to allowing families to witness resuscitation efforts. Staff described their internal conflict with this practice, as they felt its implementation was ‘*traumatic*’ for families, despite acknowledging its reported benefits in the literature:

I’ve always fought with the idea that when you are doing CPR and resuscitation, an awful lot of the research says that you should bring the family in while you’re in that process, [. . .]. I totally disagree with it. I think it’s awful like you know I certainly would never want to arrive into that space where there’s 10 people around my- somebody who I care for greatly. I- I couldn’t think of anything worse. (NP)

Significant psychological challenges for staff were also evident in participant interviews. Staff reported experiencing burnout, moral injury and mental health difficulties as a result of caring for patients and their families and their desire to provide them with a ‘nice death’. This was attributed to the often abrupt nature of death in the ICU and having to manage the family’s ‘*traumatic reaction*’ to the dying process. Staff reported being heavily relied upon to provide psychological support to the family. While staff detailed some mental health resources available to them, the presence of an ICU psychologist was described as inconsistent, despite the need for such a role being highlighted by participants; ‘*The clinical psychology role to be able to support the staff* . . . *would be a game changer in my eye if we had it across all of the critical care units*’ (NP).

### Enduring the ‘tough’ nature of dying in critical care requires better supports for staff and families

This key finding reflected several themes demonstrating a perceived need for better, more specific, and more consistent supports in critical care, especially in the context of providing a ‘nice death’. Staff recognised the ‘tough’ nature of dying and death in ICU and discussed the significant impact this environment has on staff and families. A key theme under this finding demonstrates that supports and resources for staff and families are inconsistent, insufficient, and not specific enough across ICUs in Ireland. While one participant described ‘*loads of resources*’ available to staff and families, another described the available resources as ‘*limited*’. Participants described variation in the provision of informal bereavement supports such as ICU diaries and sympathy cards across ICUs in Ireland, in addition to a lack of standardised supports for both staff and families. Staff described often signposting families to services, with a theme describing a lack of awareness of available support services for staff and families. Other themes under this key finding discussed how the COVID-19 pandemic highlighted the lack of practical resources for staff such as changing rooms, shower areas and debriefing spaces, and how it disrupted the bereavement process for both staff and families, and the ability for staff to provide a *‘*nice death*’.* While debriefings were considered a helpful support by staff in this study, their presence in both a formal and informal sense were described as inconsistent across ICUs.

Staff felt they require additional supports and resources to endure working in this challenging environment, including a need for formalised bereavement support and communication training to assist them in supporting a *‘*nice death’ for families. Staff reported that communication training would assist staff to ensure they deliver news in a clear, concise, compassionate way. As one participant reported, ‘*I don’t think I felt, and just I know from speaking to my peers [as] we’ve had detailed conversations about this, that our training has been adequate in providing end-of-life care*’ (NP).

In addition to recognising a need for better support for staff, one theme demonstrated the acknowledgement by staff that families need additional support due to the ‘tough’ nature of dying and death in the ICU. Across ICUs, staff communicated a lack or inconsistency of facilities and supports for families going through end-of-life situations and described chaplaincy as the main bereavement support provided to families in Ireland especially in the absence of psychological or bereavement counselling support. The need for better infrastructure in the ICU, with particular mention of individual family rooms was also reported. These rooms were described as a both a dedicated space for staff to discuss a patient with their families, and a space for families to grieve the death of their loved one privately. These rooms may lessen contact with the critical care environment for grieving families, which may reduce the traumatic experience associated with it. Some participants described not having family rooms, while one described theirs as

. . . somewhere to go to make a cup of tea and bring in a sandwich or to have a shower and there’s a toilet there that they can use so there is, now probably nowhere near what we need but there are some facilities there (AHP).

Suggestions to improve bereavement care included employing a bereavement liaison officer and implementing bereavement specific services such as support groups for bereaved families of ICU patients. Staff discussed how services such as these might help families to make peace with losing their loved one in ICU and provide a supportive space to grieve. The role of psychology was described as a ‘*gamechanger*’ for supporting staff and families across ICUs with one allied health professional saying, ‘*It’d be useful for coping and being able [to deal] with the prospect of losing a loved one and also what to expect in the process or even just for someone to listen*’ (AHP). An additional sub-theme highlighted the need for bereavement-specific follow-up support for families and was described as important given the ‘tough’ nature of death in ICU.

### Staff recognise the need for bereavement supports to reflect a multicultural Ireland

Bereavement supports need to better cater to the evolving multicultural diversity of Ireland. The final key finding reflected themes relating to understanding families’ needs, and how the evolving multicultural diversity in Ireland has changed staffs’ ability to do so. Themes highlight the need for bereavement supports to better cater towards the more culturally diverse population of modern Ireland. Staff expressed their desire to be culturally competent in different presentations of grief and expressed their wish to have greater awareness of other religions through cultural education, with one saying, ‘*I’m an expert coming to the area of Catholicism but other religions I could definitely do with [being] brought up to speed*’ (NP).

As mentioned previously, staff reported chaplaincy-based bereavement services as the main bereavement support available to families in Ireland throughout the dying process. While some critical care staff described this support as ‘*brilliant*’, others described the insufficient nature of this support in relation to the current multicultural, multi-religious population in Ireland, including those with no religion: ‘*The relatives rely on the chaplaincy for support . . . it isn’t great really to be honest, the support there for them*’ (NP).

Staff report they have begun to adapt how they support the process of dying, death and bereavement, particularly with regards to religious artefacts, and asking families what or who they would like to be present ‘*whatever religion they are*’, to facilitate a ‘nice death’ that is specific to the individual person.

## Discussion

This study aimed to gain insights into multidisciplinary critical care staff members’ experience of supporting the process of dying, death and bereavement in ICU, and their role related to patients and their families in this context. This qualitative interview study identified four key findings that reflected clusters of themes relating to the aim of the study.

The finding reflecting the desire to provide a ‘nice death’ implies that staff want to give patients and their families more than the routine medical care they provide at end-of-life, and not focus solely on keeping the patient comfortable. This finding suggests a more holistic approach to the dying process where staff want to go beyond providing a ‘good death’ for the patient and employ more person-centred practices, often without being taught to do so. While previous literature discusses the concept of a ‘good death’,^10,21,29^ this is distinct from the ‘nice death’ described by participants in this study. This desire among staff might be attributed to the majority representation of nursing within the sample, and related professional practices. However, practices to ensure a ‘nice death’ were described by staff in other disciplines too. Future research could further examine this concept and its implications for practice, with the aim of understanding how it is distinguished from a ‘good death’.

Recent evidence calls for better inclusion of palliative care and integration of palliative care teams in ICU,^30–32^ given the expertise in family-centred care that optimises comfort in end-of-life situations.^32^ This recommendation is in line with the most recent Guidelines for the Provision of Intensive Care Service (GPICS).^33^ The interest in incorporating palliative care into ICU was reflected in this study, as participants felt it would help to ensure providing that ‘nice death’. Furthermore, findings reflecting environmental and organisational barriers to care including resource constraints, lack of time and inadequate infrastructure are consistent with previous literature examining bereavement in critical care.^4,14,17,19^ It may be important to standardise resource provision across hospitals in Ireland, to develop ways of reducing the environmental impact of the ICU setting on the dying process.

Previous literature identified the provision of futile care,^11,12^ and discordance between staff regarding end-of-life decisions^5^ as particularly difficult challenges in ICU. These issues were reflected in the present study, where critical care staff described conflict around care decisions within the MDT. The moral distress staff can experience as a result of inefficient decision-making regarding end-of-life described in this research and previous literature highlights the need for greater clarity on this issue, and the need to ensure that end-of-life decisions are adhered to once made. Regarding resuscitation procedures, research with both critical care staff^34^ and family^35^ discussed the positive implications of family witnessing these efforts for closure and acceptance purposes. However, there is debate about the positives of this^36^ and participants in the current study expressed disagreement with this practice, despite being aware of the literature supporting its potential benefits. A possible reason for this could be the lack of follow-up with bereaved families in the ICU. Staff may not have the opportunity to explore families’ perspectives of witnessing the resuscitation efforts of their loved one at a later stage. Perhaps if follow-up were established, staff could better evaluate the impact this has on families, while training might support staff in this assessment. This finding sits in the context of debate about this issue and highlights the need to concretely establish how beneficial viewing resuscitation procedures is for families in ICU.

This study has further established in an Irish context the previous finding that working in critical care is psychologically challenging for staff.^1,5,9,10^ This recurrent theme demonstrates a need to better care for the psychological wellbeing of all critical care staff. This could include implementing scheduled formal debriefing for staff or having a professional psychologist role established across critical care settings in Ireland. As part of their role, psychologists could help to support families and staff in ICU settings in relation to death and dying, in addition to providing support for the full breadth of experiences that are known to be distressing in this environment, in accordance with the Integrated Practitioner Psychologists in Intensive Care Units Guidelines.^37^

This study reflects previous research in the U.K. examining bereavement care practices and supports^7,8,38^ and suggests that bereavement supports in ICU in Ireland are insufficient, inconsistent, and not specific enough. The lack of appropriate bereavement support may negatively impact a family’s grieving process. Future research might investigate the extent of the inconsistencies described between what bereavement support is offered to families in end-of-life situations across Ireland and find ways to improve these supports.

This study additionally reflects previous research highlighting a need for education in providing bereavement support^14,17,19^ focusing on communication skills. This implies a gap in the current education provision for critical care staff and it may be important to put greater emphasis on training in these areas. However, the desire of participants in this study to be more culturally competent in different presentations of grief and to have greater awareness of other religions through cultural education may be unique to this sample. Most participants in this study were culturally and ethnically Irish, which may have implications for the generalisability of this finding (in line with Sim’s [1998] concept of theoretical generalisability^39^) across ICUs in Ireland, and in other critical care settings. Future research might examine the perceived need for education of other cultures in ICU from a more culturally diverse critical care staff population.

### Study strengths and limitations

This study captured the perspectives of 15 staff from several disciplines working in critical care, reflecting the multidisciplinary approach in this setting. While participants ranged in ICU experience from 6 months to 23 years and were recruited through several avenues, a possible limitation of this study could be the uneven gender split in the sample. There was also a lack of cultural diversity in the sample, which does not reflect the wider critical care staff population in Ireland, which is increasingly multicultural. While the influence of religion on available bereavement supports was discussed, a possible limitation of this study could be that other aspects of the SOCIAL GRACES^40^ were not explored or reported by participants. In terms of the methods employed, data analysis was conducted in line with a recognised model, which was explicitly described, as were the steps taken to add credibility to the findings. Care was taken to ensure that the quotes provided to support the findings were reflective of the wider MDT perspectives of the sample.

### Implications and future research

The findings of this research have valuable implications for practice, education and research. This study suggests there is a need to improve ICU infrastructure and implement psychological support for both staff and families. This psychological support is in line with the most recent GPICS.^33^ Furthermore, bereavement supports and follow-up specific to death in ICU could benefit family members. Education of staff in cultural competence, and formalised bereavement and communication training were suggested by participants to improve overall service provision of bereavement support in critical care. The necessity of appropriate training was previously outlined in the NICE guidelines.^41^ In addition, formally involving the palliative care team in the ICU setting could improve the dying process for patients and their families. Future research could also examine family members’ experience of the dying process in critical care and their perspectives on supports provided, this could further guide improvements to bereavement support throughout dying and death in critical care.

## Conclusion

This qualitative research adds to the evidence base exploring perceived challenges in supporting the process of dying, death and bereavement in critical care. It also offers potential solutions to some issues, providing insight from a multidisciplinary perspective. Staff need to be supported in providing a positive experience of death for patients and their families by implementing better infrastructure, standardised bereavement support practices, psychological support for both staff and families and the addition of a bereavement support specialist to critical care teams. This reflects previous research conducted by Berry et al.^7^ in the U.K. and suggests that bereavement supports in ICU in Ireland are perceived to be insufficient, inconsistent, and not specific enough.

## Supplemental Material

sj-docx-1-inc-10.1177_17511437241308672 – Supplemental material for Exploring perspectives of supporting the process of dying, death and bereavement among critical care staff: A multidisciplinary, qualitative approach Supplemental material, sj-docx-1-inc-10.1177_17511437241308672 for Exploring perspectives of supporting the process of dying, death and bereavement among critical care staff: A multidisciplinary, qualitative approach by Elsa Joyce, Suzanne Guerin, Lindi Synman and Melanie Ryberg in Journal of the Intensive Care Society
