# Supplementary material for: Exploring perspectives of supporting the process of dying, death and bereavement among critical care staff: A multidisciplinary, qualitative approach
Source: J Intensive Care Soc. 2025 Jan 3;26(1):21–8. doi: 10.1177/17511437241308672 (PMC11699554; doi:10.1177/17511437241308672)
Supplement: sj-docx-1-inc-10.1177_17511437241308672 – Supplemental material for Exploring perspectives of supporting the process of dying, death and bereavement among critical care staff: A multidisciplinary, qualitative approach [file sj-docx-1-inc-10.1177_17511437241308672.docx]

S**upplementary Materials**

**Appendix 1; Interview Schedule**

Introduction

· Thank you for seeing me today and offering to take part in this study.

· I would like first to outline the study so that you are able to decide whether you wish to proceed further (recap information sheet).

· I have a list of topics that I want to address.

· Feel free to ask questions at any stage during the interview.

· I might make a few notes in case I want to come back to something later.

· First, are there any practical questions you have with regards to the interview?

· Okay, I’m going to begin the recording now

Topics/questions

1. Background information on the interviewee. (Explain that we are asking for the purpose of describing the sample)

· Demographics, age, gender you identify as, professional discipline

· Current and any prior relevant roles/responsibilities, training, duration in employment.

2. Identify and elicit details of staffs’ general experience of being with patients and their families during the process of dying, death, and bereavement in Ireland.

· For example:

o The role of critical care staff in the dying process in ICU.

o Their personal/specific role in supporting the dying process

o Potential differences in experience of sudden deaths or prolonged deaths

o Others . . .

3. Identify and elicit details on staffs’ general reflections and perspectives on the broad nature of supports staff provide in critical care settings in Ireland to patients who are dying and their families.

· For example

o Types of supports they perceive they give to patients

o Any specific support they themselves provide

o Others . . .

4. Identify and elicit details on aspects of current practices in bereavement support in Ireland and how it is provided to patients and their families

· For example:

o Resources available to staff in providing support to dying patients and their families

o Any bereavement/support training they may have received

o What current practices work well? What practices don’t?

o Others . . .

5. Identify and elicit details on perceived need patients and their families have for those supports

· For example

o What are the needs of the families post-bereavement in critical care?

o Do they think that these families have any different/additional needs to any other type of bereavement?

o What is useful to patients and their families? What is not?

o How important are the supports they themselves (staff) provide?

o Are there any needs of patients/families not being met?

6. Identify and elicit details on models of bereavement support that are in place in critical care settings in Ireland

7. Identify potential recommendations staff may have to improve bereavement support in Ireland during the dying, death or bereavement process in critical care settings.

· For example

o Areas where further research is needed

o Better practices in supporting dying patients and their families

o Other…

8. Identify and elicit details on models of bereavement support in the current context of the COVID19 Pandemic in critical care settings in Ireland

· For example

o Differences in service delivery during this time?

o Are there any challenges to providing this care to patients and their families that arose from the pandemic?

o Changes to how bereavement support is provided in critical care since the pandemic.

o Other…

9. Anything else that the interviewee feels has been missed and anything that they did not get a chance to discuss fully.

End of interview – thank you.

**Table 3 – Key Findings, Themes and Quotes**

| **Key Findings** | **Themes** | **Quotes** |
| --- | --- | --- |
| The critical care environment undermines staff in supporting a ‘nice death’ | Staff want to provide a ‘nice death’ to patients and their families | *“We have like a kind of a box, and we have candles in it and flowers, and we would lay that out…”*    *“The role is essentially the same in that it’s to support the patient in, with comfort and dignity so they’re as pain-free as possible and that they’re humanity is preserved”*    *“What we try to do is get rid of as much evidence of their critical care journey as you possibly can and have them laid out and have them looking as much like themselves as you can.”* |
|  |  | *“So really what you have to do is in awful lot of explanation to the family around what is happening and that takes a lot of soft work, a lot of soft communication, a huge amount of empathy, an awful lot of trying to step into their shoes”* |
|  |  | *“The family have this kind of distorted idea mainly influenced by movies and Hollywood of what withdrawing support actually is.”* |
|  |  | *“It’s all about … respecting their desires and their wishes, the patient”*    *“Particularly if they are I suppose religious or like I think it offers a lot of comfort to the family to know that there was a priest with them, you know, at the end and they got a final blessing”* |
|  | Staff have a role ensuring the comfort of the patient | *“Just make sure they’re comfortable, that the area is appropriate, that its quiet.”* |
|  |  | *“I suppose, if you could do, tap in a bit more to the palliative kind of pathway type thing like the [hospice charity name] and if they were more involved in hospital-based [work] bar just being palliative unit.”*    *“It was mad that we didn’t always include palliative care.”* |
|  | ‘Huge’ supportive role for family | *“You’re obviously looking after the patient, but you have a certain responsibility to the family as well because it is a situation that’s probably very alien to them.”* |
|  |  | *“The nurse is the key person and the most, probably, important person in getting them, in providing psychological support to the families because they are the one that is there all the time.”*    *“(Nurses) are definitely a bit more at the emotional hard point of it than some of the doctors would be.”* |
|  |  | *“So, we have a little altar, and we bring it in and there’s a nice cloth to put on it and holy water and a crucifix and there’s, you know, laminated leaflets with prayers on kind of thing.”* |
|  | Role in deciding and initiating dying process | *“We would be the ones that would initiate the process”* |
|  |  | *“What you’re trying not to do is place the responsibility of the decision on the family but at the same time trying to let them be a part of the decision-making process, you know, so it’s, it’s quite complex”* |
|  | Environmental and organisational barriers get in the way of staff providing the care they feel families deserve at EOL | *“Removing those type of anxieties away from them by enabling them to have the right infrastructure, the right comfort areas so that’s a, that’s what a good death looks like for their loved one”*    *“Now unfortunately [bereavement support nurse] is only there Monday to Friday, 9 to 5”*    *“They’re outside now, waiting on the corridor which is very… its very… its quite cold you know? And it’s not that way because we want it that way, we just don’t have the space.”* |
|  |  | *“…and the environment as well ... I don’t think you know ... the best place for end-of-life care”*    *“So, I do think those noises and things they hear around can be, can be traumatic. And there might also be other patients dying or passing away or very sick that they have to listen to what’s going on while they’re grieving.”* |
| The desire to support a ‘nice death’ poses distinct challenges for staff | Staff struggle with moral dilemmas related to dying and death in the ICU | *“You can literally allow them into resuscitation situation as well, so it, it seems to mean a lot to some people, so I do offer it but I do wonder about it sometimes, it does seem more traumatic than anything else really.”*    *“They’re very slow at stopping the vitals, the- it’s the nurse in you”*    *“So, often times as the nurse there is that moral and ethical challenge sometimes, the nurse providing the treatment might feel like its futile and that can be conflicting from like an ethical or moral perspective.”* |
|  | Staff experience of supporting the dying process differs depending on death trajectory | *“And how do you tell the family that?”* |
|  | Burnout and psychological impact of CC ‘very real’ for staff | *“The burnout is very real”*    *“Those things take their toll if you don’t talk about them”* |
| Enduring the ‘tough’ nature of dying in critical care requires better supports for staff and families | The ‘tough’ nature of death in ICU and its impact on staff demands additional support for ICU staff | *“I think because of the nature of you know, how sick they were, it’s going to be a very different to you know okay maybe they were old age, and it was kind of expected. Whereas in ICU, most of the time , it’s not an expected death.”* |
|  | Staff are sometimes not aware of the resources available to themselves or families internal/external to hospital | *“I haven’t looked into it to be honest so I’m not aware of what services are available.”*    *“I’m not aware of any particular resources that families would be given or anything.”* |
|  | Supports and resources for staff and family is inconsistent, insufficient and not specific enough | *“They have somewhere to go to make a cup of tea and bring in a sandwich or to have a shower and there’s a toilet there that they can use so there is, now probably nowhere near what we need but there are some facilities there.”*    *“Yes. There’s loads [of resources]”*    *“We’re so limited in what we have available to us”*    *“…but for bereavement in general but I don’t think there’s anything else around”*    *“I don’t think people get enough support at all, to tell you the truth.”* |
|  | Formalised bereavement support training and education could assist staff to provide a ‘nice death’ to families | *“So, there’s not- there’s nothing formal which there should be.”*    *“I don’t think I felt, and just I know from speaking to my peers, we’ve had detailed conversations about this, that our training has been adequate in providing end-of-life care.”*    *“So yeah, that little piece of education does make a difference”* |
|  | Debriefing is an important support for staff | *“I think it’s, it’s an acknowledgement that that was tough. You were in tough situ-, that was a tough circumstance, you were in a tough situation”*    *“We did have a, we had a debrief afterwards and it was, you know, and we sat down, and we spoke about it, but it doesn’t happen enough.”*    *“The fallout from the last two years is that we haven’t had the same opportunities to do, sort of, informal debriefing”*    *“The debriefing has been just brilliant”* |
|  | Families need additional support due to the nature of death in ICU | *“I would think the biggest thing we need is resources. Whether that’s resources of rooms and space, family space as well as private rooms for people to spend their last hours, and staff to support them and their families in their last hours.”*    *“A simple room where the person is at the centre of it all I think that would be so nice as well”* |
|  |  | *“I’m quoting figures off the top of my head but I think there’s about a 30% chance that a family member will be traumatised by spending time with a family member in dying in intensive care whether they pass away or not.”*  *“The families need to be offered some sort of psychological support definitely if they’re experiencing traumatic deaths in ICU”* |
|  |  | *“I think having some follow-up for that would be phenomenal in supporting them in that experience.”* |
|  | COVID highlighted the lack of resources for staff | *“There was a realisation when COVID kicked in that, ‘you know what? I’ve got nowhere to go and have a shower after I’ve worked.”*    *“This is why nurses are burnt out, even ICU nurses, this is why they are burnt out. Because there’s no, nowhere for them, bar the EPA and all that to say, to have a debriefing”* |
|  | COVID disrupted the bereavement process for families and staff | *“I think the other thing that was different was that we weren’t able to do that peer support because we didn’t have spaces to meet”*    *“With COVID when they couldn’t’ grieve properly as in laid out, have them you know have the neighbours around or whatever, I think they found that very hard to grieve”* |
| Staff recognise the need for bereavement supports to reflect a multicultural Ireland | The evolving multicultural diversity of Ireland changed staff ability to understand family’s needs | *“Now there’s such diversity like, you know?”*  *“Education on, because we are a more diverse population now, on different religions and yeah what to do, you know.”*  *“As we became multicultural, our ability to understand the needs of other people- our Indian and Filipino nursing and our African nursing colleagues have been amazing in teaching us those different aspects of what’s needed and what’s expected”*    *“I mean a booklet, definitely a booklet would be useful on different religions and their you know their cultural I suppose beliefs and, you know, what they expect I suppose or what they, what their traditions are in their, with their own religions.”* |
|  | Bereavement supports need to better cater to the evolving multicultural diversity of Ireland | *“We’ve a very good chaplaincy service, they’re brilliant”*    *“A lot of old-school, there’d be a lot of religious artifacts would be left with the patient so now, we ask. We ask ‘do you want us to have religious pieces here?”* |
